# Misrepresentation

**Published:** 1882-02

**Authors:** Ad. Lippe

**Affiliations:** Philadelphia


### MISREPRESENTATION.

Editor of The Homœopathic Physician:

Dear Sir: Permit me to draw the attention of the readers of your journal, and also of the profession in general, to a new *modus operandi* of stamping out the Hahnemannian School of the healing art, called Homœopathy; and to the way the open enemies of that school began this work by the wilful misrepresentation of the utterances of men belonging to it.

I quote from the *British Journal of Homœopathy*, October 1st, 1881, page 348: "*Drs. Chargé and Pompili represent the pure 'Hahnemannian' School, and seem to think that almost any drug may cure intermittents. The former gives indications for seventy medicines. The latter adds Zingiber to the list, but omits Cinchona and its alkaloid altogether! Both, however, beat a most undignified retreat from their position in the face of pernicious intermittents, and admit that Quinine must be given for them if the patients' lives are to be saved. The excuses they make for this inconsistency are utterly illogical; but we are glad that common sense is too much for their consistency.*"

What did Dr. Pompili say?

We find in the Transactions of the "World's Homœopathic Convention" of 1876, page 439, a paper by G. Pompili, M. D., Rome, Italy, on the "Roman Fever." He dwells first on the decided quinine mania of which nearly the whole allopathic body in Italy is suffering; he gives a frightful picture of the sufferings inflicted on credulous patients by their enormous doses of *Sulphate of Quinine*. He further says: "Young beginners in their apprenticeship to Homœopathy, whose words some indolent and doubting homœopaths echo, may ask: 'But in cases of pernicious intermittents, in which one paroxysm not controlled in time may be followed by another which may prove fatal, shall we renounce the *Sulphate of Quinine* and search for a homœopathic remedy whose success, when selected, may be uncertain?' We respond at once that, here in Rome, some parnicious intermittents occur, as already observed, though their number and frequency are greatly exaggerated. This is convenient for the doctors of the Old School, because it relieves them from thought and authorizes them to prescribe strong doses of *Sulphate of Quinine*. Besides, to what other remedy could they betake themselves in their incredulity and blindness? This good fortune of sparing brains and fatigue is naturally envied by the indolent, faithless homœopaths above mentioned. Just as we might agree to a badly selected remedy which does not correspond to the symptoms, so we may agree, in the gravest cases only, to have recourse to a remedy, which, however injurious, may sometimes avert a danger. Let us come to an understanding, then, viz., that the blame of this proceeding is not to be laid to Homœopathy, but it is to be considered only a '*testimonium paupertatis*,' as Bœnninghausen said."

*Comments.*—Did Dr. Pompili, as the *British Journal of Homœopathy* charges, beat a most undignified retreat from his position in the face of pernicious intermittents, and admit that *Quinine* must be given for them if the patients' lives are to be saved? Dr. Pompili did not, could not, beat such a retreat. Did he not make such proceedings conditional? Just as he might or would agree to a badly selected remedy which does not correspond to the symptoms, so would he agree to a remedy *Quinine*, in the gravest cases only, a remedy which *may* sometimes avert a danger. Here is the final and real question: would Dr. Pompili agree to a badly selected remedy which does not correspond to the symptoms? Not likely, we are sure he *never* would, because he does *not* belong to that class of indolent, faithless homœopaths he has so well described in his paper. Nor does he say that he, individually, ever was compelled to submit to such a humiliation; but he protests against the charge which has been brought against Homœopathy on account of this very objectionable practice of giving *Quinine* to save brains and labor. He designates it a *testimonium paupertatis*, which testimony should be granted all these indolent and faithless homœopaths who are too ignorant and too indolent to select a remedy which does correspond to the symptoms of the patient, independently of whether he suffers from a grave case of intermittent or from scarlet fever, diphtheria, or small-pox. Brains and fatigue must be spared, that is the whole upshot of the excuses offered by pretending homœopaths who fully deserve a "*testimonium paupertatis.*"

The author of the above quoted paper winds up his tirade by saying—"The excuses they make [should be he makes] for this inconsistency are utterly illogical; but we are glad that common sense is too much for their consistency."

Dr. Pompili is credited as representing the pure Hahnemannian school. Hahnemann did found a school of the healing art and named it Homœopathy—now under strict logic that school is either all right or it is all wrong: the experiment was the only possible test, and Drs. Pompili and Chargé have consistently declared their adherence to that school. The learned writer has now informed us that common sense is far superior to consistency! What a new discovery by a set of men who have spoken before of Hahnemann's imbecility: common sense dictates to them the superior logic of becoming inconsistent followers of the founder of Homœopathy; and here they exhibit before the medical profession in particular, and to the world in general, a true picture of their own imbecile inconsistency and obviously show their motives for so cruelly attacking Dr. Pompili; he is a homœopath by conviction, and that after he had honestly and intelligently made the experiment. Thereby he showed himself possessed of common sense and could not be otherwise than consistent. It is contrary to common sense, and proves inconsistency, to treat intermittents with massive doses of *quinine*. To convict Dr. Pompili of this offense and of having abandoned the principles governing Homœopathy is the great desire of a set of inconsistent men who sail under false colors that they may with impunity administer *Quinine* in massive doses, save brains and fatigue, and charge Dr. Pompili and the other true homœopaths with having endorsed their pernicious practice. Will Dr. Pompili and the large number of consistent homœopaths endorse this decided quinine-mania exhibited of late years unreservedly by professing homœopaths? Not at all. Dr. Pompili and all men belonging to the pure homœopathic school will continue to show by their practice that intermittent fevers as well as all other curable diseases are readily, promptly and mildly cured by Homœopathy under its immutable tenets; and if the pure Hahnemannians are now as capable of proving the superiority of Homœopathy over all other methods of cure as was the founder of our healing art and the early pioneers, it follows logically that these doubting and indolent men are not deserving the honorable name of homœopaths. If, by their various inconsistencies, by their increasing departures from Hahnemann's methods, they are able to found a superior school of the healing art, let them show us the proofs of their new discoveries by their superior successes. If they can do so let them drop the name of that other school under which such superior successes can not be obtained. Misrepresenting consistent men, which has become, now-a-days, a mania and prevails like an epidemic, is a poor policy; it exhibits the great weakness of a bad cause; it will not injure or hurt the consistent men who serve as a target, nor will it serve as an intimidation. Homœopathy will progress surely and will in the future, as it has in the past, not only live, but thrive on the slanders of the old school and of misled, indolent and doubting homœopaths. Yours truly,

AD. LIPPE.

Philadelphia, December 20th, 1881.

